# Editorial: Proceedings of the 2nd ISESSAH Conference 2018

**DOI:** 10.3389/fvets.2020.00052

**Published:** 2020-02-11

**Authors:** Didier Raboisson, Bouda Vosough Ahmadi, Marisa Peyre, Henk Hogeveen, George John Gunn, Jonathan Rushton

**Affiliations:** ^1^IHAP, Université de Toulouse, INRA, ENVT, Toulouse, France; ^2^The European Commission for the Control of Foot-and-Mouth Disease (EuFMD), Food and Agriculture Organization of the United Nations (FAO), Rome, Italy; ^3^CIRAD, Animal, Santé, Territoires, Risques et Ecosystèmes (ASTRE), Centre de Coopération Internationale en Recherche Agronomique pour le Dévelopement (CIRAD), TA C 22/E Campus International Baillarguet, Montpellier, France; ^4^Business Economics Group, Wageningen University & Research, Wageningen, Netherlands; ^5^Scotland's Rural College (SRUC), Edinburgh, United Kingdom; ^6^Institute of Infection and Global Health, University of Liverpool, Liverpool, United Kingdom

**Keywords:** economics, social sciences, ISESSAH, infectious diseases, biosecurity

## Introduction

Knowledge and skills in economics and social sciences are becoming increasingly significant in the animal health sector and play an important role in making sure animal health investments are people-centric (1). These skills provide a basis for decision-making processes within the livestock and animal health sectors at scales ranging from individual owners to farms, and from the livestock and food industries to regions and countries. The trend toward greater reliance on economics and social science skills reflects the complexity and variability of situations in the field, which require a whole-system approach (2)^1^. General “one size fits all” rules are not sufficient anymore; rather, insights are needed into the economic impacts of animal diseases, the profitability of potential interventions, and an understanding of the behavior of the people involved, including farmers, veterinarians, government officials, and the public. The need to take into account different views and therefore values of resources and outcomes during decision making often requires multi-criteria analysis to inform the trade-offs society faces in resource use. The optimization of societal benefits has to consider an individual's behaviors and social relationships within the disciplines of animal health management and disease control.

The International Society for Economics and Social Sciences of Animal Health (ISESSAH), established in 2017^2^, held its second conference in Montpellier, France in May 2018. The Society promotes transdisciplinary research and joined with the Innovation in Animal Health International Forum^3^ and the Economic Reasoning for Improved Animal Health Network^4^ to ensure this was achieved at the Montpellier meeting. The proceedings of this 2nd ISESSAH conference focus on how economics and social science approaches can support decision making and governance in animal disease prevention, surveillance, and control. The aim of the conference was to highlight how the principles of economic assessment and social sciences can be applied by stakeholders and leading thinkers in the field to support animal health education, research, and policy making. The 11 papers in the proceedings reflect three themes: infectious diseases, biosecurity, and alternative methods.

## Economic Assessment and Decision Analysis Applied to Infectious Diseases

Three studies highlight the usefulness of re-contextualization to improve decisions related to animal health. Montiel et al. applied the “Sustainable Livelihoods Perspective” to Mexican goat farming, demonstrating that brucellosis control offers an opportunity for small-scale goat farmers to stabilize their income and contribute to rural population welfare, ultimately reducing the likelihood of migration to the U.S. Using participatory research and interdisciplinary dialogue with Basongora pastoralists in Uganda, Chenais and Fischer highlighted how paying attention to “situated knowledge” and “embodied objectivity” improved the relevance of advice on cattle disease control. Lastly, Pramuwidyatama et al. investigated 27 measures against highly pathogenic avian influenza (HPAI) in Indonesia in 2012–2017. The animal vaccination-based HPAI mitigation strategy chosen by the government to safeguard humans from HPAI transmission had a low implementation feasibility that was attributed to insufficient collaboration among farmers.

Three other studies proposed quantitative economic evaluation to support decisions on zoonoses or national disease control programs. Thomas et al. used a food chain risk analysis model to determine the incremental cost-effectiveness ratio (ICER) of *Taenia solium* control strategies. The addition of a vaccination and treatment protocol to meat inspection (+10.3% cost) improved the ICER by 74.6%, and reduced pork industry losses from condemned meat by 66%, highlighting the potential to leverage private sector investment. Because the rationale of *Salmonella* control in pig feeds is debated, Niemi et al. carried out a cost-benefit analysis on the current Finnish control policy as compared to a reduced-control scenario. The current control policy benefits consumers, while a substantial portion of the cost is borne by feed operators; this suggests that a focus on financial responsibilities could increase acceptability of the current policy. Lastly, Gethmann et al. evaluated the German compulsory program to eradicate bovine viral diarrhea (BVD), in force since 2011, through a cost-benefit analysis between BVD control and no-control scenarios. None of the scenarios leading to complete BVD eradication was economically attractive [benefit-cost ratios (BCR) 0.64–0.94]. Only the former and the current national BVD control programs of “ear tag testing and culling” reduced BVD prevalence to 0.01%, with acceptable BCRs of 1.22 and 1.24.

## Economics and Social Sciences Applied to Biosecurity

Biosecurity is a powerful tool to manage animal diseases, from enzootic production to emerging diseases. The investments are not specific to any particular disease, and good biosecurity practices form the basis for sustainable production, yet farmers often have difficulty justifying and implementing biosecurity measures. Through a systemic approach to understanding human behavior, economic modeling, and social sciences research can assist in defining multi-disease benefits from biosecurity measures and lead to strategies that improve biosecurity compliance. Four studies in the proceedings emphasize the importance of this type of research.

The core biosecurity recommendations outlined in the U.S. Secure Pork Supply Plan include written site-specific biosecurity plans that involve the implementation of a perimeter buffer area and a line of separation. Pudenz et al. showed the complexities of biosecurity measures adoption. Their results indicate that adoption is affected by how feasible producers believe implementation of each biosecurity practice is for their operation, and on the producers' perception of risk. The authors found that implementation of one biosecurity practice was likely to increase the marginal efficacy of another biosecurity practice, such that a global approach may be useful. Merrill et al. also addressed this with a “serious gaming” approach, showing that compliance in biosecurity is influenced by the method of message delivery, increased situational uncertainty, and increased risk. Similarly, Bucini et al. developed an agent-based model that combines epidemiological dynamics and heterogeneous human decisions. Scenarios applied to porcine epidemic diarrhea virus showed that relatively small shifts (10% of the producer agents) toward a risk averse position can lead to a significant decrease in total incidence of disease. Lastly, the big-five personality traits were associated with biosecurity level as expressed by a “continuous animal hygiene index” and a “technical animal hygiene index” (Döring et al.). Interactions of personality traits with biosecurity level were demonstrated, and the results depended on production systems and rating perspectives.

## Focus on New Methods

ISESSAH promotes innovative economic and social science methods applied to animal health in order to improve animal health and welfare policies, programmes, and actions worldwide. A good example of this is the study by Barratt et al. on foot-and-mouth disease management using an innovative time series methodological framework to estimate the indirect costs of animal disease control strategies. This model takes into account how market dynamics may change following a disease outbreak, and estimates more precisely the indirect costs and wider knock-on price effects between sectors. The work by Merrill et al. and Bucini et al. are some of the first applications of “nudge theories” to animal health actors, and are based on experimental economics methods. Nudges for greater compliance with practices or that modulate risky behaviors appear to be promising approaches for animal health.

Together, the 11 papers in the 2nd ISESSAH conference proceedings provide a good overview on how different economic and social science approaches can contribute to animal health management and disease control. Complementarity among disciplines and continuous improvement in methods will support better decision making in animal health in both the short and long terms. Through the organization of annual conferences and many other initiatives, ISESSAH provides opportunities for animal health professionals to achieve wider societal benefits. Gathering our forces and competencies and focusing them on improving animal health is our organization's daily motivation.

## Author Contributions

DR drafted the editorial. MP had an advisory role and provided input at the designing stage of the Research Topic. JR, HH, BV, and GG reviewed and revised the editorial and contributed to the reviewing and editing of the papers published in the proceedings of the ISESSAH inaugural conference.

### Conflict of Interest

The authors declare that the research was conducted in the absence of any commercial or financial relationships that could be construed as a potential conflict of interest.

## Footnotes

^1^https://animalhealthmetrics.org/approach/

^2^http://www.isessah.com/

^3^https://www.alphavisa.com/isessah-innovsur/2018/

^4^https://epidec.weebly.com/
